# An Energy Efficient Sink Location Service for Continuous Objects in Wireless Sensor Networks

**DOI:** 10.3390/s20247282

**Published:** 2020-12-18

**Authors:** Cheonyong Kim, Sangdae Kim, Hyunchong Cho, Sangha Kim, Seungmin Oh

**Affiliations:** 1 Advanced KREONET Center, Korea Institute of Science and Technology Information, Daejeon 34141, Korea; cykim0807@kisti.re.kr; 2 Division of Computer Science and Engineering, Kongju National University, Cheonan 31080, Korea; sdkim.cse@gmail.com; 3 Department of Information Communication, Chungbuk National University, Cheongju 28644, Korea; hccho@chungbuk.ac.kr; 4 Department of Computer Engineering, Chungnam National University, Daejeon 34134, Korea; shkim@cnu.ac.kr

**Keywords:** energy-efficiency, mobile sinks, continuous objects, wireless sensor networks, routing protocols, location service

## Abstract

In wireless sensor networks (WSNs), detection and report of continuous object, such as forest fire and toxic gas leakage, is one of the major applications. In large-scale continuous object tracking in WSNs, there might be many source nodes simultaneously, detecting the continuous object. Each nodes reports its data to both a base station and mobile workers in the industry field. For communication between the source nodes and a mobile worker, sink location service is needed to continuously notify the location of the mobile worker. But, as the application has a large number of sources, it causes a waste of energy consumption. To address this issue, in this paper, we propose a two-phase sink location service scheme. In the first phase, the proposed scheme constructs a virtual grid structure for merging the source nodes. Then, the proposed scheme aggregates the merging points from an originated merging point as the second phase. Simulation results show that the proposed scheme is superior to other schemes in terms of energy consumption.

## 1. Introduction

The amount of damages from either disasters or accidents is influenced by the level of the prevention and countermeasures system. Especially, in case of the distant areas from the social infrastructures, such as a mountainous region and a forest area, a prevention system exerts a stronger influence then the case of a downtown area because of their low accessibility. As a promising solution for a prevention system in inhospitable areas, Wireless Sensor Networks (WSNs) have been receiving significant attention. A WSN consists of low-cost and small sensor nodes and is able to provide sensing information to remote users by self-organizing manner. These advantages enable construction of high-level prevention systems using WSNs with low-price. This paper is the part of an effort to facilitate advanced disaster prevention and environment monitoring system using WSNs. Concretely, we focus on increasing the usefulness by reducing energy consumption in communication parts. After sensor nodes are deployed in interested field, they organize a network by self-organizing manner. The information about the base station, called a sink, is embedded into sensor nodes before deployment or advertised on the network after deployment. Once an event is occurred, the sensor nodes exchange messages with each other to elect a reporting node, called a source. The source generates a data packet and sends it to the sink by multi-hop transmission. These features leverage the applications of sensor networks ranging from military surveillance to habitat monitoring [1,2].

Object tracking is one of the important applications in WSNs where sensor nodes detect where and when the object exists. There have been many researches on object tracking with sensor networks. Target objects are divided into two categories: individual objects and continuous objects. The individual objects usually have very small size comparing with the large area with sensor network deployed. An individual object is sensed by one or few sensor nodes. For example, an individual object might be an animal in habitat monitoring or an intruder in military surveillance.

**Figure 1:**
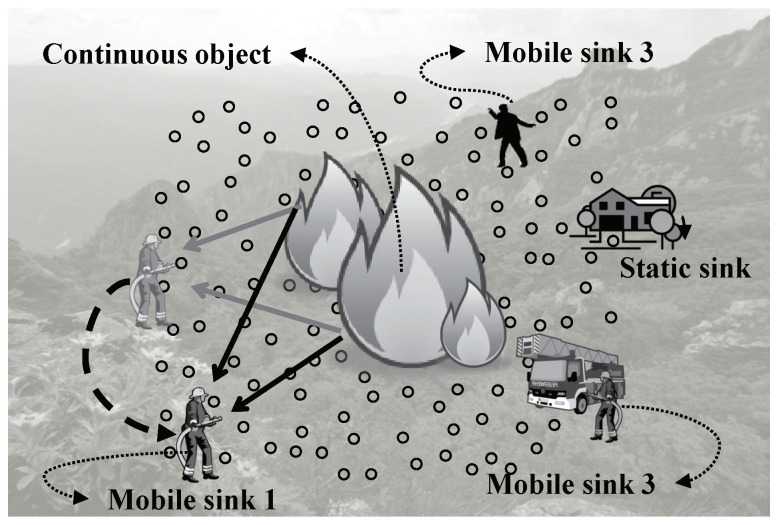
An example of continuous object tracking: Forest fire monitoring system.

In contrast with the individual object, a continuous object is a large-scale phenomenon which is originated from a specific point (e.g., a spark in the forest or a crack on the gas pipe) and spreads continuously. Then, the continuous object occupies large area and it is detected by many sensors simultaneously. The continuous object might be fire in forest fire monitoring system and harmful gas in leakage alarm system. Therefore, continuous object tracking is suitable for prevention of disasters and environment monitoring. Figure 1 shows the forest fire monitoring system as an example of the continuous object tracking. Once forest fires brake out in the sensor field, detecting information is reported to the control center. Then, the fire fighters start extinguishing the fire. During extinguishing process, latest detecting information should be reported to the control center as well as the fire fighters. The fire fighters can move to strategic locations by means of the information from the sensor network. Besides, the information is provided to the mountain climbers as well. The climbers can find fire escape routes by means of the information. In this scenario, the nodes detecting fire are sources. The fire fighters and climbers act as mobile sinks which are mobile communication entities whereas the control center is a static sink which is located at fixed location.

In the scenario described above, the applications should satisfy the following requirements:

- Sink mobility support: In case of a static sink, it is possible that providing sensor nodes with its location information as a priori knowledge because its location is fixed. On the other hand, a mobile sink has to notify its location to the network periodically because it moves continuously.
- Reducing data report cost: Because a continuous object is a very large phenomenon and continuously diffuses, the detecting information generated by a number of sources brings considerable communication overhead for reporting data. The overhead might shorten network life time. To prolong network lifetime, the data report cost have to be reduced.

The existing continuous object tracking studies [3,4,5,6,7,8,9,10,11] focus on energy-efficient reporting. The primary issue in the studies is reducing the number of reporting nodes without distortion and loss of detecting information. The studies exploit data aggregation by clustering or election of representative by local cooperation. However, the existing studies are inapplicable to the above-described scenario in terms of supporting sink mobility because they have not dealt with mobile sinks. The satisfaction of sink mobility means a mobile sink can collect data from sources despite the mobile sink moves continuously. To collect data, a mobile sink has to periodically notify its current location to the sources because it moves continuously.

**Figure 2:**
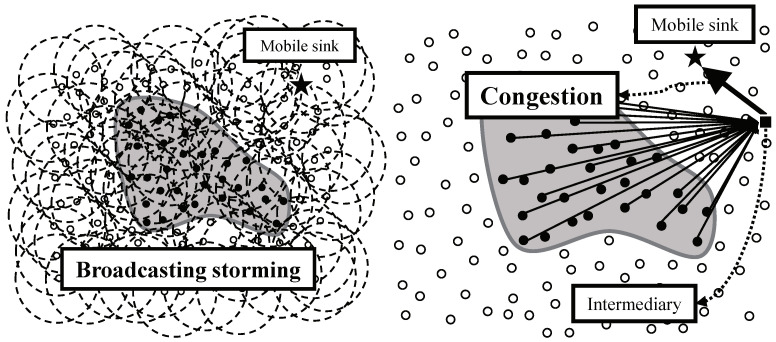
Intuitive solutions for supporting sink mobility: (**left**) Flooding-based approach and (**right**) Intermediary-based approach.

Intuitively, the most simple approach is the flooding. A mobile sink could notify its current location by flooding the location to whole network like Figure 2 left. However, periodic flooding for location notification of a mobile sink brings the waste of bandwidth and energy of the network called the broadcasting storming problem [12]. Instead of using flooding, a mobile sink can receive data from an intermediate agent like Figure 2 right. Assuming the agent is more powerful than sensor nodes and always knows the mobile sink’s current location (e.g., a static sink can be the agent), the mobile sink can receive data from the agent without notifying its location to sources. The sources just forward data to the agent. However, in this case, a large number of data packets pass the area between the agent and the mobile sink. This concentration lead to both congestion and hot-spot problem [13,14].

**Figure 3:**
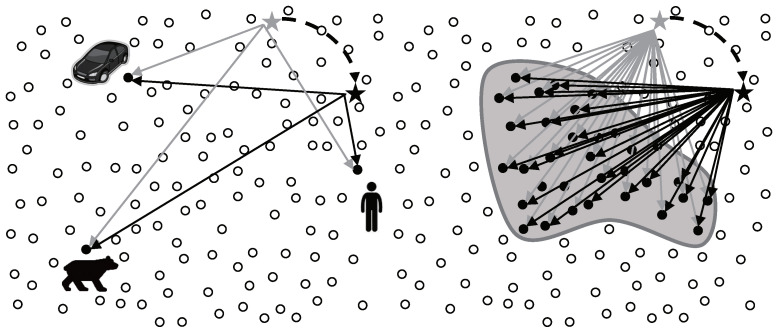
Location update of a mobile sink in individual object tracking and continuous object tracking: (**left**) Individual location update and (**right**) Integrated location update.

To avoid the problems in flooding-based or intermediary-based approach, the adoption of existing studies for supporting sink mobility is a reasonable alternative. One of the most important issues in sink mobility support studies is how to notify the current location of a mobile sink. However, the studies are not suitable in continuous object tracking because they have dealt with not continuous objects but individual objects. In case of tracking individual objects like Figure 3 left, the studies provide the location updates between a mobile sink and each source. Likewise, in case of tracking a continuous object like Figure 3 right, the studies provide the location updates between a mobile sink and each source detecting the continuous object individually. The individual location updates lead to significant energy consumption, and consequently, shortening the network lifetime. From the view of the continuous object tracking, the location updates between a mobile sink and a large number of sources are unnecessary because the sources detecting a continuous object are contiguous each other.

Our aim is to design an energy efficient sink mobility support scheme for continuous object tracking. The previous continuous object tracking researches focus on reducing communication cost of reporting data from a number of sources without considering of sink mobility. Unfortunately, the existing studies for supporting sink mobility are inappropriate in continuous object tracking because they have dealt with individual objects. To facilitate advanced continuous object tracking, the sink mobility support scheme being suitable for continuous object tracking is required. The main problem in existing sink mobility support studies is the individual location updates to the sources detecting a continuous object. The location updates are essential for supporting sink mobility, but lead to wasting energy. Our previous work focuses on the connection the origin point to each sinks [15]. We extended the work for energy efficiency. To achieve energy-efficiency, we propose Origin-based Continuous Object Tracking scheme (OCOT). The OCOT provides integration of the location updates by using origin-based structure thereby reducing the overhead for supporting sink mobility.The main contributions of our work are as follows:

- We intensively investigated the energy efficiency in continuous object tracking with mobile sink by analyzing the location update and data report to prevent hot spot problem and to prolonging network lifetime.
- The proposed scheme provides the integrated location update by mobile sink and distributed data report for reducing overall energy consumption and mitigating hot spot problem.
- Our simulation results in various environment provides the analytical insights for revealing the radical limitation of the existing schemes and the key principles of the proposed scheme thereby promoting the future developments.

The rest of the paper is organized as follows. We present the related work in Section 2. The proposed scheme, OCOT is described in Section 3 and the experiment result of OCOT is showed in Section 4. Finally, We conclude this paper in the Section 5.

## 2. Related Work

WSN is used for various applications such as forest fire monitoring, gas leakage monitoring, and radiation monitoring because it is easy to use and provides cost-efficiency and flexibility compared to wired networks [16]. For example, Kadri et al. proposed a forest fire detection system using WSN that consists of three functional blocks: fire detection, sending alerts, and intervention [17]. In the system, the wireless sensors detect ambient temperature and the value of CO2. When the sensor data exceed a predefined threshold, the alerts are sent to the base station thereby leading to the intervention of forest guards. Therefore, for building the disaster management system, continuous object tracking is the fundamental functional requirements of the WSN.

In wireless sensor networks, several protocols to detect and track continuous object have been developed. They focus on how to efficiently detect the continuous object and reduce the amount of reports about continuous object toward a static sink. On the other hands, many protocols to support sink mobility have been proposed with the aim of disseminating data to mobile sinks. In this chapter, we briefly introduce continuous object tracking schemes and evaluate the protocols for sink mobility support with features of continuous object tracking.

According to [13,18], sink mobility is the cause of balanced energy consumption of sensor nodes inside a network. Data sensed by distributed sensor nodes are transmitted through multi-hop communications of intermediate nodes between a source and a sink. The nearer sensor nodes to a sink receive, forward, and propagate a more amount of data if the location of the sink is fixed. The nearer sensor nodes therefore consume more energy and more rapidly die out. In other words, their lifetime is more shortened than that of nodes far from the sink. Consequently, died sensor nodes around the sink can not play their role of communications and sensor, and functions of the network can also stop. This phenomenon is called, the hot-spot problem [13,14]. On the contrary, sensor nodes around sinks do not keep consuming energy if sinks change their location frequently. Such the hot-spot problem can be overcome by adopting mobile sink. The sink mobility also might be adopted by the nature of the deployed application [19]. For example, in the scenario in Figure 1, both the fire fighters and climbers act as mobile sinks.

**Figure 4:**
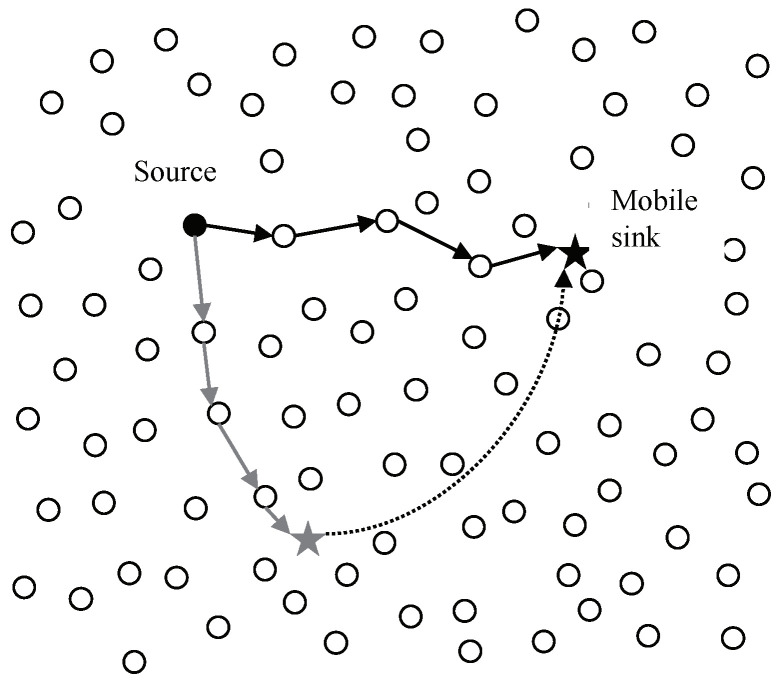
Data dissemination route before and after the mobile sink moves.

A number of WSN researchers have interested in supporting sink mobility [19]. They assume that sinks might be either stationary or mobile in the sensor field and mobile sinks might move around a sensor network with random mobility [20]. In other words, a static sink is a stationary object providing sensor nodes with its fixed location information as a priori knowledge, while a mobile sink is a moving object frequently updating its location to the sensor nodes. Figure 4 shows change of route for data dissemination before and after a sink movement.

In such a context, the difficulty for sensor nodes is to efficiently update the mobile sink location to report sensor data. In many researches, overlaying a virtual infrastructure over the physical network has been investigated as an efficient strategy for an effective data dissemination to mobile sinks [21,22]. They use the concept of virtual infrastructure, which acts as a rendezvous area for collected data from sources and location update messages from mobile sinks. The nodes belonging to the structure designated to store reported data from sources during the absence of a mobile sink. After the mobile sink updates its current location to the structure, the designated nodes report data to the sink. Virtual infrastructures are divided into two categories: *source-based structure* and *independent structure*.

### 2.1. Source-Based Structure

Some researches on the sink mobility exploit source-based structure. In the approach, a source detecting an event initiates construction of a structure. The structure provides the route tending to the source on whole network. A mobile sink might loads location update message thereby delivering the message to the source. The source reports detecting data to the sink through the reverse route. The most representative is Two-Tier Data Dissemination (TTDD) model for large-scale WSNs [21].

**Figure 5:**
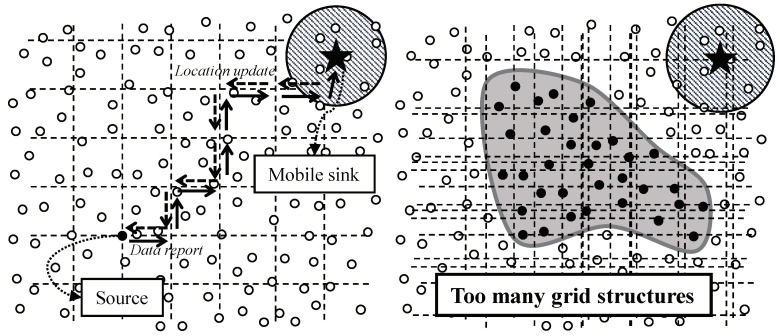
Examples of object tracking in TTDD: (**left**) Individual object tracking and (**right**) continuous object tracking.

In TTDD, once an event is detected, the source pro-actively builds a virtual grid structure, as shown in Figure 5 left. The source chooses itself as the starting grid point in the grid structure and then sends notification to its four adjacent grid points of the structure. The grid point nodes receiving the notification message become the downstream nodes of the source. The notification process continues until the grid structure is completely constructed on whole network. A sink propagates its location to the closest grid point node by local flooding. The grid point node sends the message to its upstream node. This location update continues until the source receives the message. Once the source receives the location update message from one of its downstream grid point nodes, the source sends out data to this grid point node. The data report process continues until the data reach the mobile sink. In individual object tracking, TTDD supports sink mobility without both flooding and intermediary.

On the other hands, in continuous object tracking, TTDD might causes a serious dissipation of energy. As mentioned above, in TTDD, the grid structure is constructed by a source as the initiator. In continuous object tracking, a number of sources are generated because a continuous object is large and diffusing continuously. Therefore, the sources initiate the construction of grid structures respectively like in Figure 5 right. The overhead to construct grid structures dramatically increases in proportion to the number of sources. The construction overhead might shorten the network lifetime due to the excessive consumption of energy.

### 2.2. Independent Structure

Meanwhile, several researches exploit independent structure for supporting sink mobility [22,23,24,25,26]. The researches assume that each sensor node knows its geographic location as well as the network geographic boundaries. A node position can be obtained through Global Positioning System (GPS) or some virtual coordinate system [27,28]. Eligibility of nodes comprising the structure is easily performed based on this geographic information. Thus, the overhead for building the virtual infrastructure is avoided. The location of independent structure is known as a priori knowledge to sensor nodes and sinks so all nodes including both sensors and sink might easily access it. The most representative is A Line-Based Data Dissemination protocol for wireless sensor networks with mobile sink (LBDD) [22].

**Figure 6:**
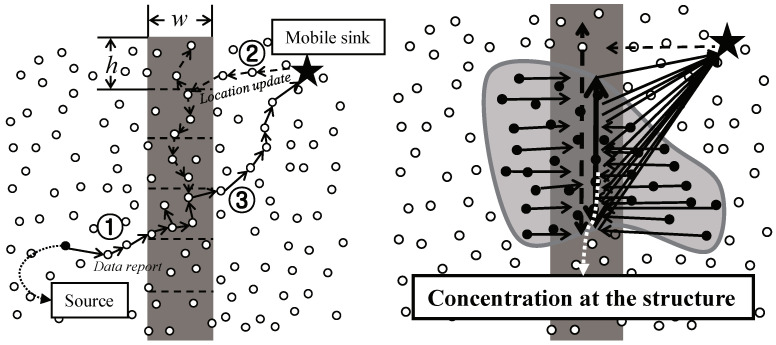
Examples of object tracking in LBDD: (**left**) individual object tracking and (**right**) continuous object tracking.

In LBDD, the independent structure is vertical virtual line which divides the sensor field into two parts, as shown in Figure 6 left. The line has *w* wide and consists of *h* sized groups. The line is placed in the center of the sensor field so that sensor nodes and a mobile sink might easily access it. The nodes within the line are inline−nodes, while the other nodes are referred to as ordinary nodes. This line acts as a rendezvous region for data storage and lookup. When an ordinary sensor node detects an individual object and generates new data, it forwards the data to the nearest inline-node(① in Figure 6 left). The inline-node shares the data with the other inline-nodes in its group. In order to collect data, a mobile sink sends a query including its current location towards a horizontal direction(② in Figure 6 left). The first inline-node which receives the query propagates it to both vertical directions along the line until it reaches the inline-node storing the data. The data is then sent directly to the sink(③ in Figure 6 left). Even though LBDD has the possibility of detouring, it is very simple and efficient protocol for supporting sink mobility in individual object tracking.

However, in continuous object tracking, LBDD might lead to a serious concentration of messages. Inherently, all messages generated in the sensor field, including data from sources and queries from sinks, pass through the line area like in Figure 6 right. The sensor nodes on the structure receive, forward, and propagate a more amount of messages because the structure is fixed as a certain shape. The sensor nodes therefore consume more energy and more rapidly die out. In other words, their lifetime is more shortened than other nodes. Consequently, died sensor nodes on the structure do not play their role of communications and sensor, and functions of the network can also stop. Namely, the hot-spot problem is occurred.

Existing continuous object tracking studies focus on reducing reporting cost considering a static sink. To support sink mobility, the adoption of existing sink mobility support researches is a reasonable solution. The researches provide sink mobility efficiently in individual object tracking by overlaying virtual structures. However, in continuous object tracking, they cause critical problems shown as following Table 1. In continuous object tracking, source-based structure consumes enormous energy for constructing the structures proportional to the number of sources. On the other hands, independent structure constructs one structure so it consumes constant energy for constructing the structure. Therefore, independent structure is appropriate in terms of the construction cost for structures. Meanwhile, independent structure might lead to the hot-spot problem because the all messages generated in the sensor field are passing through the structure. In contrast, the messages in source-based approach are distributed to each structure so source-based approach might avoid the hot-spot problem. From this, we obtain following structure design principles for continuous object tracking. First, the structure should be constructed regardless of the number of sources. Second, the dissemination routes for messages should be decentralized.

## 3. Proposed Scheme

### 3.1. Overview of the Scheme

A mobile sink should notify its current location to sources to collect data from the interested data. Generally, the location update message is directly delivered by unicasting. Namely, the location update cost increases proportional to the number of sources. So, in continuous object tracking, the location update consumes an amount of energy because a continuous object is detected by a number of sources. In energy constrained WSNs, reducing the cost of sink location update should be considered.

**Figure 7:**
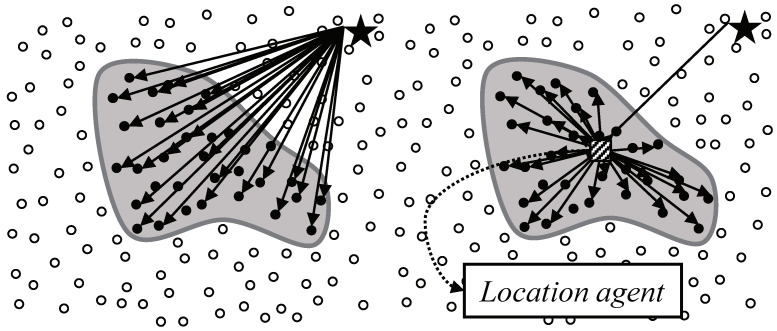
Location updates to the sources detecting a continuous object: (**left**) Individual location updates and (**right**) Integrated location updates.

**Figure 8:**
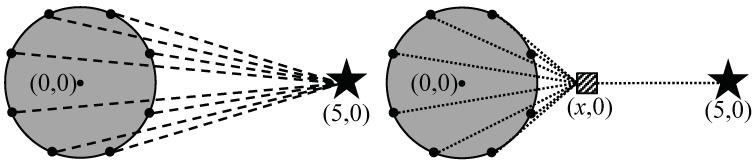
Mathematical models of individual location update (**left**) and integrated location update (**right**).

In this context, the regional locality of sources bring up the possibility of integrating location updates. In order to reduce the location update cost, we divide the location update with the following two steps: the first step is from the mobile sink to a location agent and the second step is from the agent to sources. Figure 7 shows individual location updates and integrated location updates by a location agent. Intuitively, it seems that the integrated location update is efficient.For clarification, we modeled the individual location update and integrated location update on two-dimensional plane as shown in Figure 8. The center of the continuous object is at the point (0,0) and its radius is 1. The black dots on the perimeter of the continuous object are sources. The black star indicates a sink located at point (5,0) and the hatched square indicates a location agent. In WSNs, the communication cost between two nodes tends to be proportional to the distance between them. Therefore, we assume that the sum of dashed lines is the communication cost of individual location updates, whereas the sum of doted lines indicates the cost of integrated location update using the agent. While the location of the agent varies along the x-axis, the communication cost of the individual location update is not changed. We defined the cost value *R* for presenting the efficiency of the integrated location updated compared to the individual location update as

(1) $$R=\frac{\sum_{i=1}^{n}\sqrt{{\left(x-x_{i}\right)}^{2}+y_{i}^{2}}+\left(5-x\right)}{\sum_{i=1}^{n}\sqrt{{\left(5-x_{i}\right)}^{2}+y_{i}^{2}}}$$

where *x* is the x-coordinate of the location agent, $\left(x_{i},y_{i}\right)$ is the location of the boundary points, and *n* is the number of boundary points (i.e., in Figure 8, n=8).

**Figure 9:**
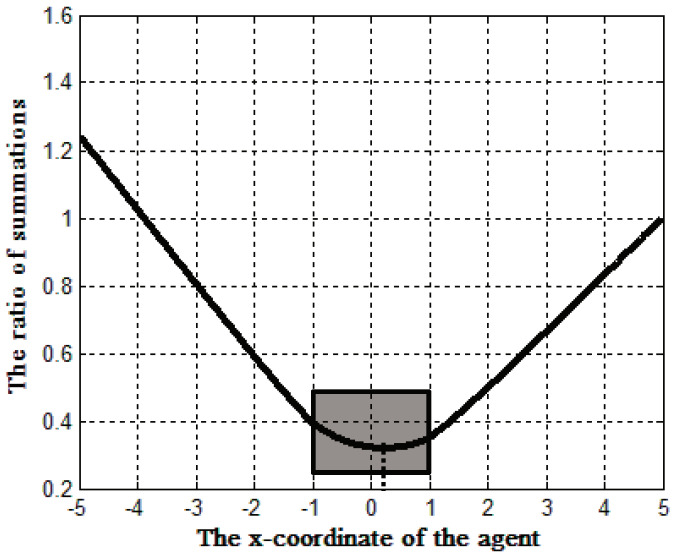
The efficiency of using a location agent.

Figure 9 shows the change of *R* according to the x-coordinate of the agent where the gray rectangular indicates the area within the continuous object. In the graph, the lower value of *R* means that the location agent is at the more efficient integration point. Here, there are two important observations. First, relatively row communication cost can be guaranteed (i.e., under 0.4 times of the individual location update) when the location agent is within the continuous object. The cost of the integrated location update tends to decrease as the agent is closer to the center of the continuous object whereas the value increases sharply as the agent far from the continuous object. Second, the location of the agent should be fixed and unique. In Figure 9, the x-coordinate of the agent resulting the lowest value of *R* is approximately 0.1 (i.e., the point indicated by the dotted line). It means the most efficient location of the agent is not deterministic but vary according to the shape of the continuous object and the location of the sink. However, calculating the most efficient location is extremely difficult because it has to consider both the location of a mobile sink and the locations of all sources in real time. Moreover, the location update process for the agent is needed if the agent’s location can be changed. To summarize, we derive the following requirements about an agent from above observation.

- An agent should be within targeted continuous object.
- An agent should be at fixed and unique location.

We select the origin point of a continuous object as an agent. Generally, a continuous object tends to be originated at specific point and to diffuse slowly. With the features, designating the origin point as the location of an agent completely satisfies above requirements. First, the origin point is located within a continuous object because the continuous object diffuses toward its surroundings. Second, the origin point is fixed although the continuous object changes its shape and size. Additionally, there is no need to calculate the location of the origin point because it is surely detected by a specific source. With the origin point, we exploit overlaying virtual structure for supporting sink mobility. The structure should satisfy the following design principles: construction of structure regardless of the number of sources and decentralization of messages for avoiding hot-spot problem.

We exploit the origin-based structure for supporting sink mobility. The brief introduction of our design is as followings. The origin-based structure delivery location update messages from mobile sinks to the origin point. The node on the origin point is responsible for forwarding the messages to sources. The sources receiving the location update message report data to the sink’s location in the messages directly. The origin-based structure is constructed regardless of the number of sources. Moreover, the direct data reporting prevents concentration of messages of the structure. With this, our design satisfies above principles. Additionally, we exploit clustering to reduce data report overhead.

### 3.2. Origin-Based Continuous Object Tracking(Ocot)

We assume for the design of OCOT as followings:

- A two-dimensional sensor field is covered by a large number of homogeneous sensor nodes which communicate with each other through short-range radios. So, long distance message delivery is achieved by multi-hop transmission. All sensor nodes in the sensor field are aware of their own locations through either the GPS [27] or other location systems [28].
- The sensor nodes are periodically awoken and observe the targeted object. Once a stimulus appears or the message is forwarded at specific geographical location, the sensors surrounding it collectively process the signal and one of them becomes the source of the stimulus or the destination of the location.
- The scheme uses simple greedy forwarding [29] as underlying forwarding method.

#### 3.2.1. Construction of Origin-Based Grid Structure

**Figure 10:**
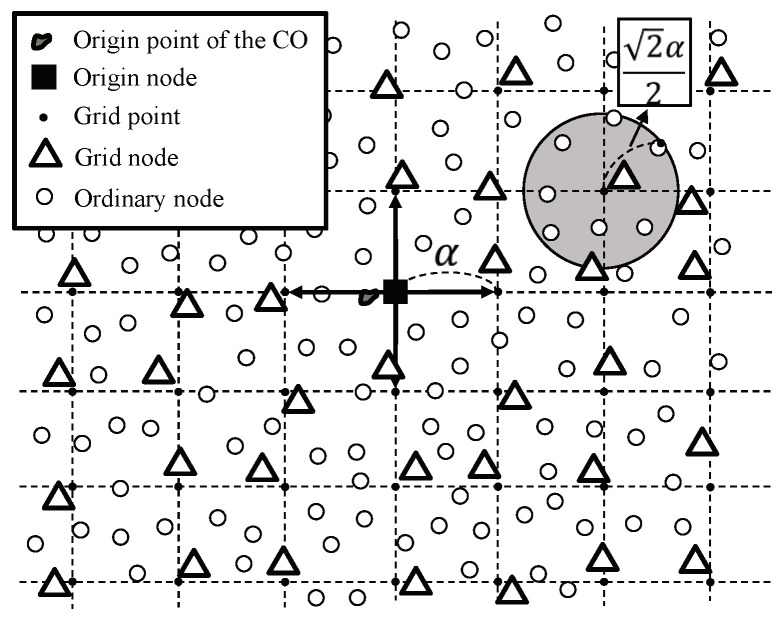
Construction of the origin-based grid structure.

When a continuous object occurs at the origin point, the first source node becomes the origin node that starts the construction of grid structure. Figure 10 shows the grid construction process where α is the grid size. The origin node sends *grid construction messages* to the four neighboring grid points. When the location of the origin node is $L_{s}=\left(x,y\right)$, the grid points are $L_{p}=\left(x_{i},y_{i}\right)$ such that:

(2) $$x_{i}=x+i\cdot \alpha ,y_{i}=y+j\cdot \alpha ;i,j=\pm 0,\pm 1,\pm 2,\ldots$$

The messages spread the whole network and the node closest to each grid point becomes the grid node. A grid node keeps the information about the grid point $L_{p}$ and upstream grid point. Duplicated messages from different neighboring grid nodes are dropped so a grid node has one upstream grid point. Then, a grid node becomes a bluster head and organizes a cluster by broadcasting a cluster construction message. The cluster size $\frac{\sqrt{2}}{2}\cdot \alpha$ is selected for thorough cluster construction. A sensor becomes a cluster member by sending its ID to the cluster head and ignores the cluster construction messages from other grid nodes.

Figure 10 shows a grid for the origin node *O* indicated with a black square, and its virtual grid structure. The black dots indicate grid points and the white triangles around each grid point are the grid nodes. The gray area indicates a cluster. After the grid structure and clusters are completely built in the sensor field, all sensor nodes become to know their cluster heads, to whom they should deliver their detection information. The cluster head receiving data from ordinary nodes becomes boundary cluster head and has responsibility for data reporting to sinks.

#### 3.2.2. Boundary Detection and Tracking

**Figure 11:**
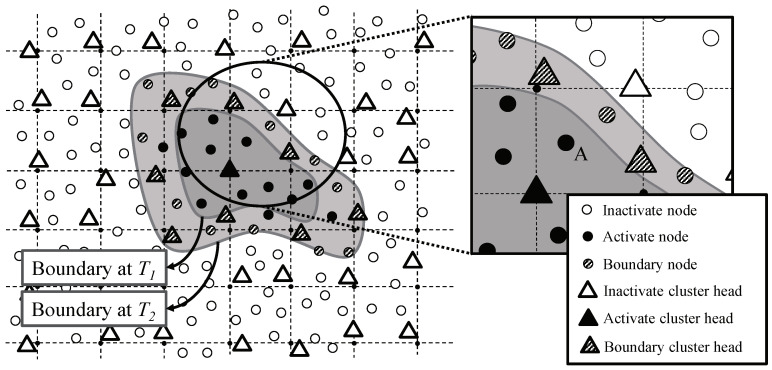
Boundary detection and tracking in OCOT.

**Figure 12:**
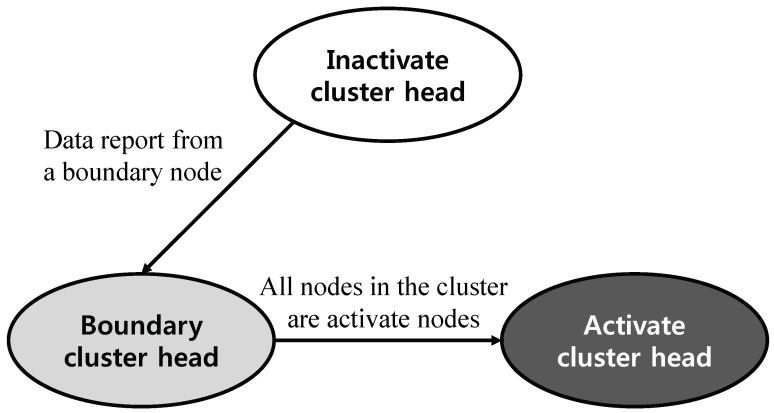
The state transition diagram of cluster heads.

A continuous object gets a great number of sensor nodes activated so it is more efficient to gather detection information only from boundary nodes rather than all activated sensor nodes. Figure 11 illustrates tracking a boundary of a continuous object where black, hatched, and white cluster heads/nodes are activate, boundary, and inactive status respectively. Figure 12 illustrates the state transition diagram of cluster heads. When a sensor node detects a continuous object in its sensing range, it communicates with one-hop neighboring sensor nodes to ask them about whether they detected the continuous object. If the sensor node has one or more one-hop neighboring sensor nodes with no detection of the continuous object, then it acknowledges that it becomes a boundary node. Boundary nodes have responsibility to report the detection information to its cluster head. Otherwise, a sensor node does not report the detection information even it is activate node, since the boundary of the continuous object already moved through its local area. For example, node *A* in Figure 11 was a boundary at time $T_{1}$ so it reported the detection information to its cluster head. Over time, the boundary has moved. At time $T_{2}$, node *A* is activate node because its all neighbors detect the continuous object, so it does not report data to the cluster head. The number of boundary nodes *K* can be estimated by calculating boundary area. For example, the continuous object has circular shape,

(3) $$K=\rho \left(1-\frac{\pi {\left(R-r\right)}^{2}}{\pi R^{2}}\right)$$

where ρ is the node density, *R* is the radius of the continuous object, and *r* is the communication range of sensors. If *R* is 100 m and *r* is 15 m, the 27.75% of detecting sensors become boundary nodes.

#### 3.2.3. Location Update and Data Report

**Figure 13:**
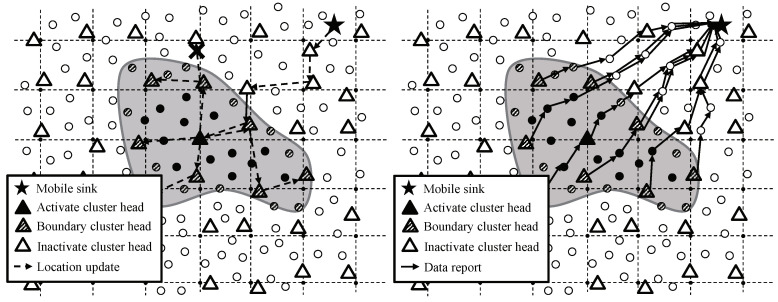
Location update and data report in OCOT: (**left**) Location update and (**right**) Data report.

As an integrated location update scheme, in OCOT, the location update message from the mobile sink is delivered to the origin node through the grid structure as shown in Figure 13 left. The mobile sink sends a location update message to 1-hop neighboring sensor nodes. Because the nodes aware their cluster head, the location update message is delivered to closest cluster head. The cluster head forward this message to its upstream grid point, eventually the message reaches the origin node. The origin node is responsible for passing the location update messages to the boundary cluster heads. The origin node delivers the message to its four neighboring grid points. The location update message is propagated by the same way with grid construction messages. The difference is that the location update message is not propagated to all grid points, but to boundary and cluster heads. Unlike location update massage, data reporting does not use the grid structure like in Figure 13 right. The boundary cluster head receiving a location update message sends collected data to the location in the message directly. This separation between data reporting and location updating contributes to avoidance of concentration to the grid structure. The movement of a mobile sink during the message and data are delivered is filled by progressive foot-print chaining [30].

## 4. Performance Evaluation

We evaluate our Origin-based Continuous Object Tracking (OCOT) through computational simulation with existing studies: TTDD [21] and LBDD [22]. The purpose of simulations is verification that OCOT is suitable for continuous object tracking supporting sink mobility in terms of energy-efficiency and network lifetime rather than existing studies.

### 4.1. Metrics and Simulation Environment

We choose two metrics to analyze the performance of OCOT. The total energy consumption is defined as the communication (i.e., transmitting and receiving) energy the network consumes during a simulation round. The total energy consumption means efficiency of message delivery. The idle energy is not counted since it depends largely on the message generation interval and does not indicate the efficiency of massage delivery. Thedistribution of energy consumption is the map including the location of each node and used energy of the node. We might deduce the possibility of the hot-spot problem in each scheme from the distribution of energy consumption. We divide the network into 20 × 20 cells and observe the average used energy of the nodes in each cell. We implement OCOT in MATLAB [31]. The default simulation environment is summarized in the Table 2. The simulation network space is 500 m × 500 m square. The number of nodes is 1000 and they are uniformly deployed. The transmission of each node is omnidirectional. The transmission range and sensing range are 20 m. The sensor node’s transmitting and receiving consumption rates are 20 mW and 15 mW, respectively. A mobile sink sends a location update message to sources every 5 s. Sources generate data every 5 s.

**Figure 14:**
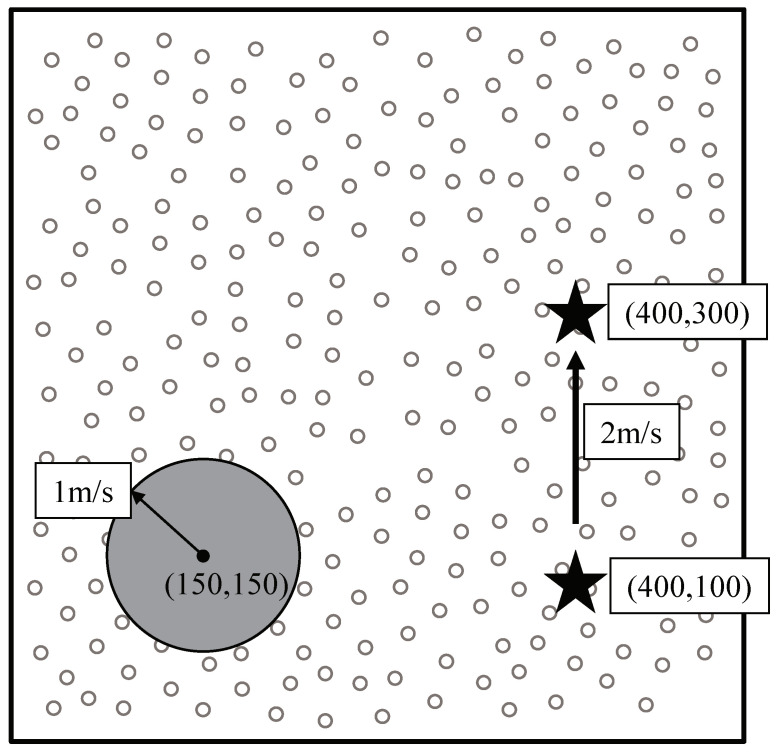
Tracking scenario.

Unfortunately, comparison with existing studies is not reasonable because they focus on sink mobility support in individual object tracking. So, we assume that TTDD and LBDD have exploited DCS [3] for tracking continuous object. In modified TTDD, the sources organize clusters dynamically, and cluster heads construct grid structures. Similarly, in modified LBDD, cluster heads report data to the line. The maximum number of sources in each cluster in modified TTDD and LBDD is set as 5. A cell size in TTDD is 50 m. The width and the group size in LBDD is 50 m, respectively. A cell size in OCOT is 50 m. Figure 14 shows a continuous object tracking scenario. A continuous object is originated at point (150, 150). The continuous object spreads circularly and its radius increases to 1 m/s. The initial location of the mobile sink is (400, 100). The sink moves to point (400, 300) at a speed of 2 m/s during each simulation round.

### 4.2. Total Energy Consumption

**Figure 15:**
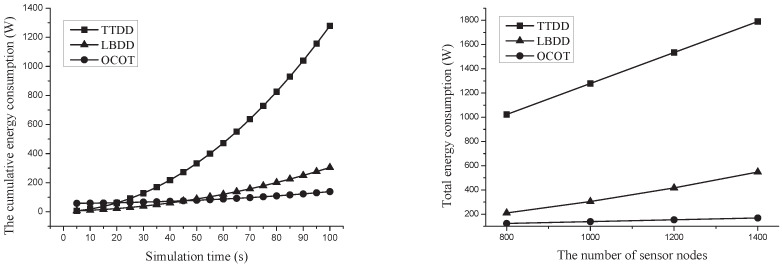
Comparison of total energy consumption according to simulation time (**left**) and the number of sensor nodes (**right**).

Figure 15 left shows the cumulative energy consumption. In TTDD, each cluster head constructs grid structure respectively. TTDD consumes little energy at early of simulation because a few sources detect the continuous object and a few cluster heads is elected. However, the energy consumption greatly increase by the time since the number of sources increases. On the other hands, the energy consumption of LBDD and OCOT much less than in case of TTDD because LBDD and OCOT construct one structure regardless of the number of sources. Though LBDD consumes much less energy, the energy consumption of LBDD draws a slow and exponentially rising curve because the reported data from cluster heads are shared into receiving group of inline-nodes. The sharing overhead increases as the number of report increases. OCOT consumes more energy than others at early of simulation since the overhead for constructing a grid structure and clusters at whole network while TTDD and LBDD comsume small energy for simple grid structure and line structure construction respectively. However, OCOT consumes much less energy at later of simulation because integrated location update and direct data report. The individual location update of TTDD and LBDD comsume much more energy.

Figure 15 right shows the total energy consumption according to the number of sensor nodes. In this simulation, we vary the number of sensor nodes from 800 to 1400. The number of sensor nodes means the average density of nodes per unit area because the network size is unchanged. We adopt simple method for selecting boundary nodes so the number of boundary nodes increases as the node density of nodes increases. The total energy consumption of TTDD rises sharply and it means that TTDD is heavily influenced by the number of nodes because of the grid structure construction overhead being proportional to the number of boundary nodes. The total energy consumption of LBDD increases slowly and it is caused by the data sharing overhead of inline-nodes while the energy for constructing line structure is not affected by the number of nodes. This shows that the grid construction overhead in TTDD is more influenced by the number of boundary nodes than the data sharing overhead in LBDD. On the other hand, OCOT is barely affected by the number of boundary nodes. Actually, the location update overhead of OCOT increases as the number of boundary nodes increases but it is relatively low compared to either the grid construction in TTDD or data sharing overhead in LBDD.

**Figure 16:**
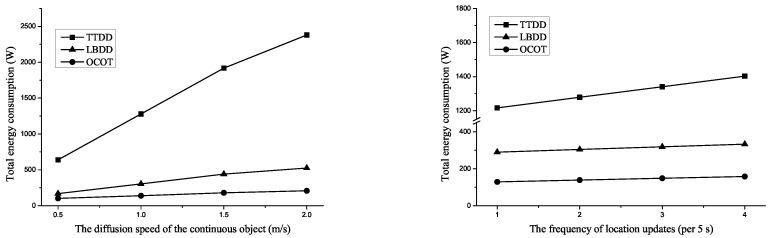
Comparison of total energy consumption according to the diffusion speed of the continuous object (**left**) and the frequency of location updates (**right**).

Figure 16 left shows the total energy consumption when the diffusion speed of the continuous object is 0.5, 1.0, 1.5, and 2.0m/s. A continuous object diffusing at high speed might occupy larger area than one diffusing at low speed at same time. Namely, the diffusion speed influence the growth rate of the number of sources. Figure 16 left is similar in the tendency to Figure 15 right. The total energy consumption of TTDD more sharply increases than others and the total energy consumption of LBDD is relatively higher than that of OCOT. The difference is shown in Figure 15 right when the diffusion speed is 2. Commonly, the gradients of each graph slightly decrease. It caused by the size of the network and the location of the origin node in the simulation setting. When the diffusion speed is less than 1.5, the perimeter of the continuous object is within the network. However, in case of the speed is 2, the perimeter increases over the network since the radius of the continuous object passes over the boundary of the network.

Figure 16 right shows the total energy consumption according to the frequency of location updates which indicates the agility to the mobility of the sink. We vary the frequency of location updates from 1 to 4 per 5 s. As the frequency increases, the influence of the location update to the total energy consumption increases. Therefore, Figure 16 right indicates the location update efficiency of each scheme. The energy consumption of TTDD increases most sharply because the location update messages are propagated to each source individually. The energy consumption tendencies of both LBDD and OCOT are similar but considering that the location update messages in LBDD are propagated to not sources but a few inline-nodes, the location update in OCOT is more efficient than LBDD because the location update in OCOT are propagated to each boundary cluster head. This integrated location update reduces the energy consumption for location updates.

### 4.3. Distribution of Energy Consumption

**Figure 17:**
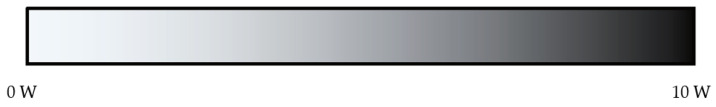
The meaning of the depth of color.

The distribution of energy consumption indicates the operational efficiency and existence of hot spot problem. Therefore, we can tangibly estimate overall energy efficiency and network lifetime. We divide the 1000 m × 1000 m network into 20 × 20 cells and observe the average used energy of the nodes in each cell. Figure 17 shows what the color in each cell means. On all of the simulations, the node, which transmitted the largest number of messages, consumed the energy less than 10 W. Therefore, we postulate 10 W for a maximum value of the total energy consumption at each sensor node. According to Figure 17, white cell consumed 0 W and black cell consumed 10 W averagely.

**Figure 18:**
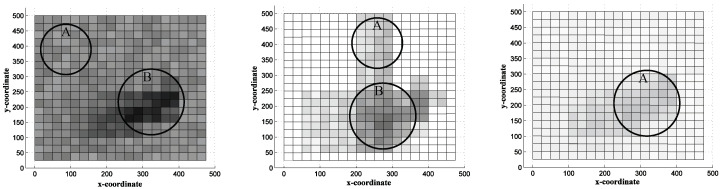
Distribution of energy consumption in TTDD (**left**), LBDD (**center**), OCOT (**right**).

Figure 18 left shows the distribution of energy consumption in TTDD. In Figure 18 left, most cells are gray even the cells which have no relationship with tracking process, such as area A. It means that most nodes wasted energy regardless of tracking process and it is because of the individual grid construction of each source. Moreover, the area B is almost black which is located between the origin point of the continuous object and the mobile sink’s moving path. It is because a lot of data reports are concentrated into the area B thereby shortening the network lifetime.

Figure 18 center shows the distribution of energy consumption in LBDD in which the most cells are white because there is no operations being performed in the area. However, the unnecessary energy consumption is occurred at area A because the inline-nodes share location update messages of the sink. In addition, the area B is deep gray because the inline nodes within same group share data from sources. In other words, the closest group to the sink is share a lot of data from the sources. In addition, the inline node, which receives a location update message from the mobile sink, reports data to the sink directly. Namely, the responsibilities for sharing and reporting data are concentrated at area B.

Figure 18 right shows the distribution of energy consumption in OCOT in which the most cells are closely white. The cells that are not related sensing the continuous object and data report consumes energy only for grid construction and clustering in contrast with TTDD and LBDD. In addition, even the area A that is between the continuous object and the moving path of the sink is light gray. The integrated location update and direct data report of OCOT disperse messages to other area which result in prolonging network lifetime.

## 5. Conclusions

In this paper, we propose a novel continuous object tracking scheme, Origin-based Continuous Object Tracking (OCOT), which provides energy-efficient sink mobility support in wireless sensor network. OCOT integrates location updates from a mobile sink using regional locality of sources detecting a continuous object. Also, OCOT exploits distributed data report to avoid the hot-spot problem. Additionally, the reactive and static clustering in OCOT increases the efficiency of location update and data report. Simulation result proves that OCOT is greatly efficient in terms of the total energy consumption and distributional energy consumption. Especially, OCOT reduces at least 49% of energy compared to the existing scheme during 100 s of continuous object tracking process. Under the same simulation setting, in addition, the over 7.25% and 1.25% of sensors consumed over 25% of the energy budget in the existing schemes and OCOT respectively, which suggests that the enhanced network lifetime of OCOT.

## Figures and Tables

**Table 1:** Evaluation about sink mobility support schemes for continuous object tracking.

Category	Construction Cost for Structures	Concentration of Messages
Source-based structure	Proportional to the number of sources	Distributed to each structure
Independent structure	Constant	Concentrated on the structure

**Table 2:** Default simulation environment settings.

Parameters	Values
Network size and # of sensor nodes	500 m × 500 m and 1000 nodes
Transmission range	Omnidirectional 20 m
Transmitting/receiving power consumption rates	20 mW /15 mW
Frequency of location update and data report	Every 5 s
